# *Palerasnitsynus* gen. n. (Trichoptera, Psychomyiidae) from Burmese amber

**DOI:** 10.3897/zookeys.130.1449

**Published:** 2011-09-24

**Authors:** Wilfried Wichard, Emma Ross, Andrew J. Ross

**Affiliations:** 1 Institut für Biologie, Universität zu Köln, Gronewaldstrasse 2, D-50931 Köln, Germany; 2 Department of Natural Sciences, National Museums Scotland, Chambers Street, Edinburgh EH1 1JF, Scotland

**Keywords:** Fossil Trichoptera, fossil insects, aquatic insects, fossil taxonomy, palaeoenvironment

## Abstract

*Palerasnitsynus ohlhoffi* **gen. et sp. n.** is described fromBurmese amber of late Albian (Lower Cretaceous) age. This is the first record of the family Psychomyiidae from Burmese amber, and the earliest fossil record of the family. The genus *Palerasnitsynus* **gen. n.** differs from all other known psychomyiid genera by the absence of fork III in the forewings.

## Introduction

Trichoptera are very rare in Burmese amber and previously only two species, *Burminoptila bemeneha* Botosaneanu, 1981 (Hydroptilidae) and *Wormaldia myanmari* Wichard & Poinar, 2005 (Philopotamidae), have been named. Burmese amber from Myanmar is late Albian (late Lower Cretaceous) in age (Ross et al. 2010). Within this paper we describe the new genus *Palerasnitsynus* based on a male and a female specimen. The new species *Palerasnitsynus ohlhoffi* is described from the male specimen. This taxon constitutes the first record of the family Psychomyiidae from Burmese amber and the earliest fossil record of the family.

Only seven fossil psychomyiid species have been described so far: *Arkharia oblimata* Sukatsheva, 1982 comes from Upper Cretaceous of south-eastern Siberia, originally placed in family Philopotamidae but belonging to the Psychomyiidae (Li and Morse 1997). The extinct psychomyiid *Trichopterodomus leonardi* Erikson, 1983 is described from larval retreats from the Paleocene of North Dakota. Five fossil species of the extant genus *Lype* Mclachlan, 1878 are known from Eocene Baltic amber (Wichard et al. 2009).

## Taxonomy

### Family Psychomyiidae Kolenati, 1859

#### Palerasnitsynus gen. n.

urn:lsid:zoobank.org:act:FD7212F5-2A0A-41AF-834B-B429CEF89188

http://species-id.net/wiki/Palerasnitsynus

##### Type species.

*Palerasnitsynus ohlhoffi* sp. n.

##### Etymology.

The new genus is named after Prof. Dr. Alexandr P. Rasnitsyn on the occasion of his 75^th^ birthday, in recognition of his extensive work on Palaeoentomology.

##### Diagnosis.

Genus *Palerasnitsynus* gen. n. differs from all extant genera (*Paduniella* Ulmer, 1913, *Psychomyia* Latreille, 1829, *Psychomyiella* Ulmer, 1908, *Lype* Mclachlan, 1878, *Padangpsyche* Malicky, 1993, *Tinodes* Curtis, 1834, *Trawaspsyche* Malicky, 2004, *Eoneureclipsis* Kimmins, 1955, and *Zelandoptila* Tillyard, 1924) and the extinct genus (*Arkharia* Sukatsheva, 1982) by the absence of apical fork III in the forewing.

##### Description.

Head: Ocelli absent. Antennae filiform, almost as long as forewings; each scapus only slightly longer than each pedicel and individual flagellomeres; flagellomeres cylindrical. Maxillary palps 5-segmented in both sexes, terminal 5^th^ segment flexible, annulated, and at least twice as long as each of the other segments. Labial palps 3-segmented with flexible, annulated, long terminal segments.

Thorax: Mesoscutum with one pair of setal warts. Legs with tibial spurs 2/4/4. Wings elongate, rounded at apex, hind wings narrower than forewings. Forewing subcosta (Sc) simple, terminating into costa (C) at about three quarters the length of wing. Radius R_1_ simple; R_2+3_ unforked, R_4+5_ forking into R_4_ and R_5_ (fork II) close to potential crossvein r; discoidal cell (Dc) short. Media M_1+2_ unforked, M_3+4_ forking into M_3_ and M_4_ (fork IV); crossvein m indistinct; median cell (Mc) apparently open. Cubitus Cu_1_ bifurcated into Cu_1a_ and Cu_1b_ (fork V); Cu_2_ simple; crossvein m-cu apparently present, closing thyridial cell (Tc). Forewings with forks II, IV, and V.

#### Palerasnitsynus ohlhoffi sp. n.

urn:lsid:zoobank.org:act:7DBC53F5-0678-4639-8D36-C698DA87655C

http://species-id.net/wiki/Palerasnitsynus_ohlhoffi

Figs 1 [Fig F2] –3

##### Holotype.

Male embedded in Burmese amber, late Albian, Myanmar, deposited in the Staatliches Museum für Naturkunde Stuttgart (ex coll. Rainer Ohlhoff, Saarbrücken). The holotype is well preserved in slightly cloudy amber. The forewings cover the hindwings in dorsal view; the hindwing venation is difficult to analyze due to the overlapping hind wings and forewings. The male genitalia are visible in ventral view. The antennae and legs are completely preserved but the left mid leg is broken.

##### Etymology.

The species is named after Rainer Ohlhoff, who kindly made the fossil available for this study.

##### Diagnosis.

The only species of the genus *Palerasnitsynus* which differs from all other known psychomyiid genera by the absence of apical fork III in the forewings. Length of male 1.9mm (intraspecific variation not known).

##### Description.

Length of male (tip of head to tip of abdomen) 1.9 mm; forewing length 1.9 mm; hind wing length 1.7 mm.

Head: Eyes large and bulbous; ocelli absent; antennae filiform, almost as long as forewings, with cylindrical scapes, pedicel and flagellomeres. Maxillary palps 5-segmented, segment 1 short, segment 2 longer than segments 1 and 3, as long as segment 4, segment 5 flexible, annulated, twice as long as segment 4. Labial palps 3-segmented with long terminal segments, nearly as long as segments 1 + 2.

Thorax: Mesoscutum with pair of setal warts. Tibial spurs 2/4/4. Forewings: Subcosta (Sc) simple, Radius: R_1_ simple, R_2+3_ unforked, R_4+5_ forked at cross-vein r; discoidal cell short. Media: M_1+2_ simple, M_3+4_ forked. Crossvein m indistinct, median cell (Mc) apparently open. Cubitus: Cu_1_ forked; Cu_2_ unforked. Crossvein m-cu apparently present, defining thyridial cell (Tc). Forewings apically rounded; venation with apical forks II, IV and V. Light-coloured hindwing venation indistinct. Rs apparently forked into R_2+3_ and R_4+5_; forks I and II apparently absent. Crossvein r apparently absent. M branched, M_1+2_ unforked, M_3+4_ indistinct.

Genitalia: In ventral view, sternite IX with sinusoidal apical margin, bearing pair of two-segmented gonopods; coxopodite short, broad at basis; harpago long. Harpagones regularly curving mesad, widening at apex. Coxopodites with row of strong hairs along median margins; each coxopodite with brush of short, dark setae at apex. In dorsal view two small black spines about half as long as gonopods, visible through hyaline wings. External structures of male genitalia indistinct in lateral and dorsal view.

**Figure 1.**
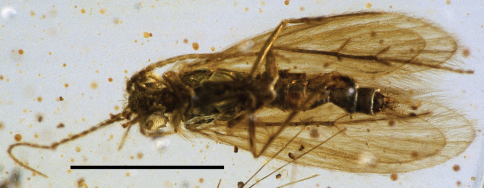
*Palerasnitsynus ohlhoffi* sp. n. holotype (male) in ventral view. Scale bar 1mm.

**Figure 2. F2:**
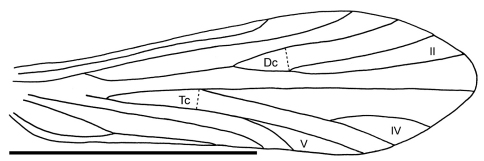
*Palerasnitsynus ohlhoffi* sp. n. male right forewing. Scale bar 1mm.

**Figure 3.**
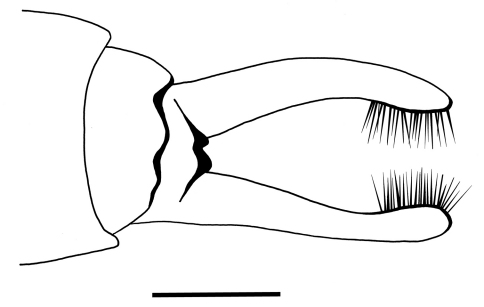
*Palerasnitsynus ohlhoffi* sp. n. male genitalia in ventral view. Scale bar 0.1mm.

#### Palerasnitsynus sp. female

Figs 4 –5

##### Material.

Female imago in Burmese amber, late Albian, Myanmar; deposited in National Museums Scotland, Edinburgh; specimen no. G.2010.20.35 (ex Scott Anderson Coll.).

##### Description.

Length of female (tip of head to tip of abdomen) 2.7 mm; forewing length 2.57 mm.

Head: Length 0.23 mm, width 0.47mm; filiform antennae each with 28 segments (including basal antennal segment), basal antennal segment twice as wide as other segments and twice as long as first segment; eyes large and bulbous, ocelli absent; maxillary palps 5-segmented, terminal segment flexible, annulated, and long (0.31mm), and nearly three times longer than each of the other segments (of similar length to each other); labial palps 3-segmented, also with long, flexible and annulated terminal segment.

Thorax: Mesoscutum with setal warts. Forelegs not easy to see; however, each may have 2 short spurs at end of tibia; mid legs each with 2 long spurs at end of tibia, 1 long preapical spur and possibly the base of a second; hind leg with 2 long spurs at end of tibia and 2 long preapical spurs. Tibial spurs: 2?/4?/4.

Wings with short fringe hairs on the anterior margin and long fringe hairs on posterior margin. Left forewing length 2.57mm; Sc dark and simple, terminating about three-quarters of length of anterior margin; R_1_ simple; Rs branching twice, first branch originating at mid-wing, R_2+3_ simple, R_3+4_ branched (= fork II); discoidal cell (Dc) present (crossvein faint); M branching twice, first branch originating at mid-wing, M_1+2_ simple, M_3+4_ branched (= fork IV); Cu branching distally (= fork V); anal veins not visible. Left hindwing apex obscured; Sc dark and simple, terminating about three-quarters of length of anterior margin; R_1_ simple, Rs branching once; crossvein r present, discoidal cell present; R_4+5_ connected to M_1+2_ by the crossvein r-m; M branching twice, M_1+2_ ending with short apical fork III, M_3+4_ simple; Cu branching once (= fork V), connected to M by the oblique crossvein m-cu closing thyridial cell apically; anal veins not visible. Forewings each with apical forks II, IV, and V; hind wings each with apical forks III and V.

Genitalia: Length 0.3mm. Typical long and simple ovipositor present; narrow cleft medio-ventrally and a pair of short apodemes apically.

**Figure 4.**
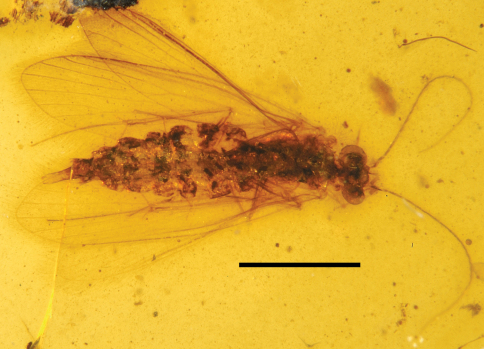
*Palerasnitsynus* sp. female in ventral view. Scale bar 1mm.

**Figure 5.**
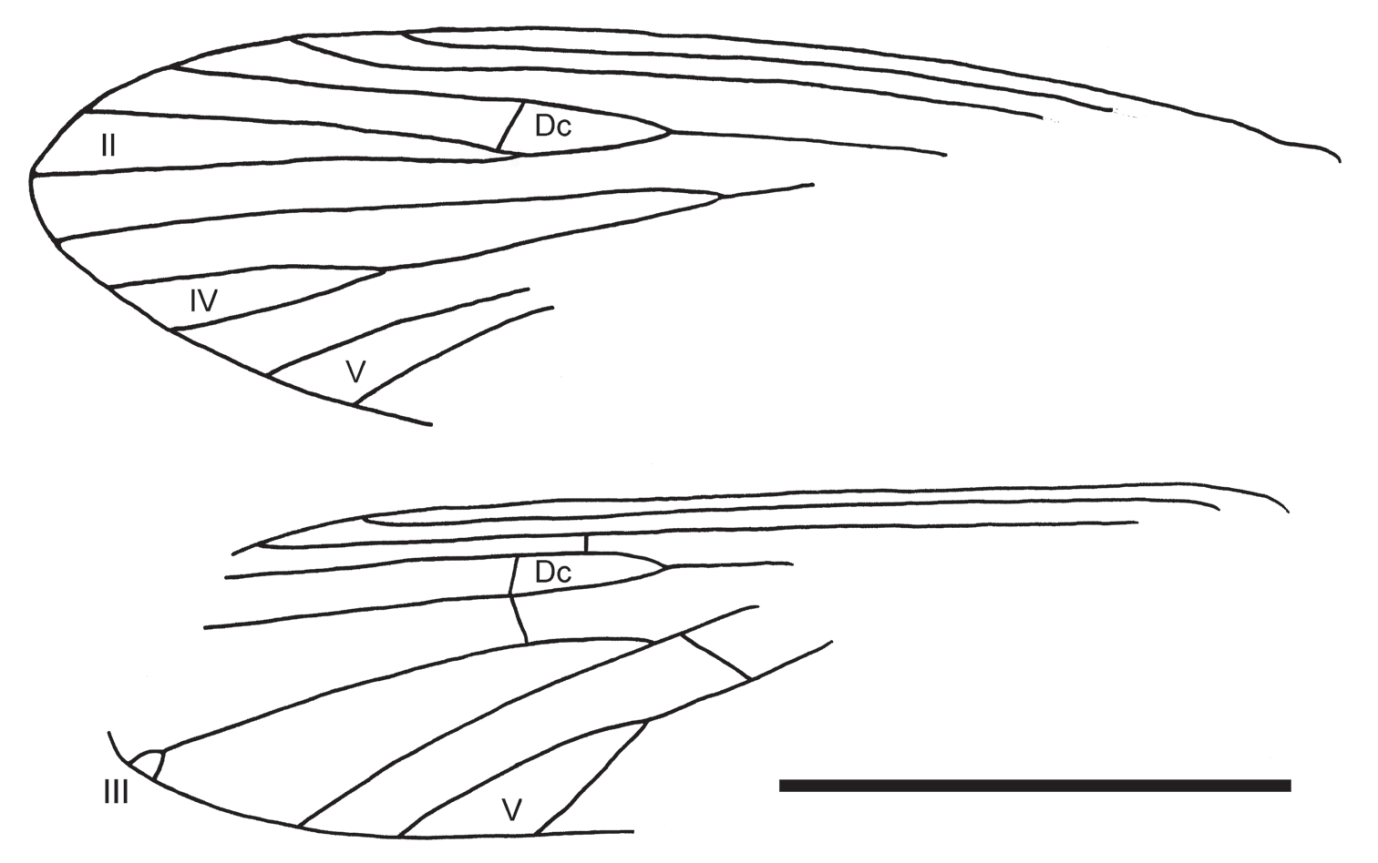
*Palerasnitsynus* sp. female left forewing (part) and left hind wing (part). Scale bar 1mm.

##### Remarks.

Due to the characteristic features (ocelli absent; maxillary palps 5-segmented and with terminal segment flexible and annulated; mesoscutum with one pair of setal warts; and tibial spurs probably 2/4/4), the female probably belongs to the Psychomyiidae. Due to the absence of forewing apical fork III, the specimen belongs to the genus *Palerasnitsynus* gen.n. The female and *Palerasnitsynus ohlhoffi* sp. n. agree in the forewing venation and differ in the hind wing; the discoidal cells are closed and short forks III are present in the female hind wings, but both traits are absent in *Palerasnitsynus ohlhoffi* sp. n. There is no evidence to identify this female specimen as *Palerasnitsynus ohlhoffi* sp. n.

## Discussion

The diversity of insects in Burmese amber is high compared with that of other Cretaceous ambers. Ross et al. (2010) listed 228 arthropod species in Burmese amber, but aquatic insects are rare both in number of individuals and number of described species. There are currently two described species of mayflies (Ephemeroptera) (Sinitshenkova 2000; McCafferty and Santiago-Blay 2009), one species of damselfly (Odonata) (Poinar et al. 2010) and an undescribed stonefly (Plecoptera) figured by Grimaldi et al. (2002). True water bugs and water striders are represented by only one species of the family Hydrometridae (Andersen and Grimaldi 2001). More frequent are amphibious flies (Diptera) in the families Ceratopogonidae, Chaoboridae, Chironomidae, Corethrellidae, Culicidae, Limoniidiae, Psychodidae, Tanyderidae and Tipulidae (total 28 described species, see Ross et al. 2010). Only three species of caddisflies (Trichoptera) have been described from Burmese amber: *Burminoptila bemeneha* (Hydroptilidae), *Wormaldia myanmari* (Philopotamidae) and *Palerasnitsynus ohlhoffi* gen. et sp. n. (Psychomyiidae).

The low diversity of aquatic insects in Burmese amber might reflect that the late Albian palaeoenvironment had few freshwater habitats. This contrasts with Eocene Baltic amber diversity, comprising 25% amphibious aquatic insects (Wichard et al. 2009). However, the exploration of Burmese amber and its inclusions is continuing and the total diversity is presently uncertain. Extant Trichoptera of Southeast Asia (with Thailand as geographical centre) have been studied in a monograph by Malicky (2010). In this monograph, about 260 species of Hydroptilidae, 180 species of Philopotamidae and 150 species of Psychomyiidae are described today, which contrasts the single fossil species of each family known in Burmese amber.

## Supplementary Material

XML Treatment for Palerasnitsynus

XML Treatment for Palerasnitsynus ohlhoffi

XML Treatment for Palerasnitsynus
